# Integrative Approach for Designing Novel Triazole Derivatives as α-Glucosidase Inhibitors: QSAR, Molecular Docking, ADMET, and Molecular Dynamics Investigations

**DOI:** 10.3390/ph17020261

**Published:** 2024-02-19

**Authors:** Oussama Abchir, Meriem Khedraoui, Hassan Nour, Imane Yamari, Abdelkbir Errougui, Abdelouahid Samadi, Samir Chtita

**Affiliations:** 1 Laboratory of Analytical and Molecular Chemistry, Faculty of Sciences Ben M’Sik, Hassan II University of Casablanca, Casablanca 7955, Morocco; oussamaabchir12@gmail.com (O.A.); meriemkhedraoui5@gmail.com (M.K.); a_errougui@yahoo.fr (A.E.); 2 Department of Chemistry, College of Science, United Arab Emirates University (UAEU), Al Ain P.O. Box 15551, United Arab Emirates

**Keywords:** diabetes mellitus, molecular docking, ADMET, QSAR, dynamics simulation, triazoles

## Abstract

**Abstract:**

In response to the increasing prevalence of diabetes mellitus and the limitations associated with the current treatments, there is a growing need to develop novel medications for this disease. This study is focused on creating new compounds that exhibit a strong inhibition of alpha-glucosidase, which is a pivotal enzyme in diabetes control. A set of 33 triazole derivatives underwent an extensive QSAR analysis, aiming to identify the key factors influencing their inhibitory activity against α-glucosidase. Using the multiple linear regression (MLR) model, seven promising compounds were designed as potential drugs. Molecular docking and dynamics simulations were employed to shed light on the mode of interaction between the ligands and the target, and the stability of the obtained complexes. Furthermore, the pharmacokinetic properties of the designed compounds were assessed to predict their behavior in the human body. The binding free energy was also calculated using MMGBSA method and revealed favorable thermodynamic properties. The results highlighted three novel compounds with high biological activity, strong binding affinity to the target enzyme, and suitability for oral administration. These results offer interesting prospects for the development of effective and well-tolerated medications against diabetes mellitus.

**Dataset License:**

License under which the dataset is made available (CC0, CC-BY, CC-BY-SA, CC-BY-NC, etc.)

## 1. Introduction

Diabetes mellitus (DM) is a chronic metabolic disorder characterized by hyperglycemia, which can be caused by reduced insulin action, inadequate insulin synthesis, or both [1]. Hyperglycemia typically aggravates the disease burden of diabetes mellitus by contributing to the development of different macrovascular complications like peripheral and autonomic neuropathy, an increased incidence of atherosclerosis, cerebrovascular diseases, neuropathy, nephropathy, and retinopathy [2].

The main feature of diabetes mellitus (DM) is thought to be accompanied by several symptoms including polyuria, polyphagia, weight loss, and blurred vision.

According to the IDF Diabetes Atlas (International Diabetes Federation), 436 million people worldwide were estimated to have diabetes in 2021; by the end of 2045, that number might potentially increase to 700 million [3,4].

The onset of diabetes mellitus is linked to diverse lifestyle factors, including smoking, excessive alcohol consumption, inadequate physical activity, and comorbid conditions such as dyslipidemia and hypertension. Additionally, genetic predisposition, stress, and obesity contribute to the risk of diabetes [5], while enzymes like alpha-glucosidase and amylase play a role in breaking down sugars into glucose and maltose, contributing to the increase in blood glucose levels, ultimately resulting in hyperglycemia and posing challenges to the proper functioning of insulin [2].

The dual inhibition of α-glucosidase and α-amylase enzymes is the most desired strategy for combating the permanent effects produced by type 2 diabetes [6]. α-glucosidase is one of the main enzymes that breaks down carbohydrates, and it is found in the brush borders of the intestine and helps break down complex oligosaccharides into simple monosaccharides [7].

By delaying the absorption of carbohydrates in the small intestine, alpha-glucosidase inhibitors effectively prompt the pancreas to release an appropriate amount of insulin. Their antihyperglycemic effect is modest, as they do not directly impact insulin secretion but rather impede the function of alpha-glucosidase in breaking down complex sugars. However, it is crucial to recognize that alpha-glucosidase inhibitors, represented by Miglitol, Acarbose, and Voglibose, are associated with various adverse side-effects, such as nausea, bloating, and flatulence [8,9].

A wide range of biological activities have attracted the attention of academia and industry to 1,2,4-triazoles and their fused heterocyclic derivatives, which are a favored structure among nitrogen-containing heterocyclic compounds [10]. Numerous clinically available, therapeutically significant drugs have been found to have the 1,2,4-triazole core such as Sitagliptin, Voriconazole, and Fluconazole [11,12].

A triazole is a ring with five members that consists of three nitrogen atoms and two Carbon atoms connected by alternating π-bonds. C_2_H_3_N_3_ is the chemical formula for this heterocyclic compound. Triazoles possess structural characteristics that make them pharmacologically active, including a moderate dipole characteristic, the capacity to form Hydrogen Bonds, ion–dipole interactions, π–π stacking, cation–π interactions, the hydrophobic effect, van der Waal forces, stiffness, and stability [13].

A variety of 1,2,4-triazole derivatives were synthesized in 2020 by Emmanuel Oloruntoba Yeye et al. [14], who also evaluated the compounds’ IC_50_ value for experimentation. The principal objective of this work is to discover new 1,2,4-triazole compounds that have encouraging action against α-glucosidase. We specifically used a 2D-QSAR study on these derivatives to build molecular models that can be used to design novel triazole derivatives and anticipate their biological activities before their synthesis [15,16].

## 2. Results and Discussion

### 2.1. QSAR Model Analysis and Validation According to the OECD Principles

Using the GA-MLR approach, this study produced a number of molecular models with descriptors in the range of (4–6). The best model was chosen based on its statistical characteristics, which indicate the robustness, strength, and consistency of the generated model. The model selected, which includes the descriptors AATSC8s, VE3_Dzs, nHsOH, CIC1, and RotBFrac as listed in Table 1 and Table 2, satisfied all of the evaluation criteria. These included the leave-one-out cross-validation coefficient (Q^2^_LOO_ = 0.633)**,** the R-squared coefficient of determination (R^2^= 0.767), the root-mean-squared error (RMSE = 0.082), the coefficient adjusted for degrees of freedom (R^2^_adj_ = 0.712), and the coefficient of determination for the test set (R^2^_test_ = 0.649).

The results of the Y-randomization test showed that none of the random trials could match the original model, as indicated in Table 1. The lesser values for R^2^ and Q^2^ on each iteration, and their averages (R^2^_YS_ = 0.195 and Q^2^_YS_ = −0.341) suggest that the developed QSAR models are not based on random correlations.

**Figure 1:**
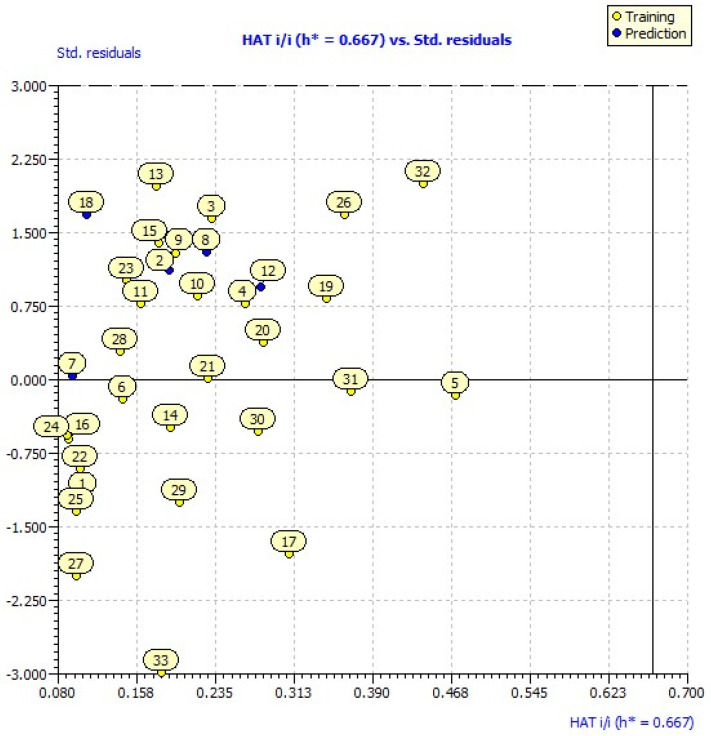
William’s plot of the developed model.

The applicability domain (AD) of the model was determined to define the chemical space of set compounds, and it aids in estimating the uncertainty in the prediction of a particular compound based on how similar it is to the training compounds. In this regard, William’s plot was employed, considering predictability using three standard deviations and leverage levels below the critical leverage. Therefore, the prediction of a modeled response using QSAR is applicable only if the compound being predicted falls within the AD of the mode. The evaluation’s findings demonstrated that neither the training set nor the test set had response values that were outside of the range of responses. The leverages of all the compounds are less than the leverage threshold value of h* = 0.667 (Figure 1), and their standard deviations are all within the ±x range (x = 3) [14].

### 2.2. Design of Novel Compounds

A prior investigation by Emmanuel Oloruntoba Yeye et al. assessed the ability of a set of 33 synthetic chemicals to inhibit α-glucosidase. According to the findings, compounds 14, 16, 20, 21, 25, 27, 28, and 33 showed action that was comparable to that of the well-known α-glucosidase inhibitor Acarbose. The analysis of these compounds consistently revealed the presence of halogen elements, amino groups, and/or nitro groups.

It was discovered that compounds with nitro, amino, or halogen components showed more inhibitory potential, while triazole derivatives with hydroxy groups exhibited less inhibitory potential.

Using the best-selected model and this information as a foundation, this study attempted to create novel drugs with enhanced α-glucosidase inhibitory action. A higher pIC_50_ value against α-glucosidase in comparison to the series’ most active compounds was the aim.

We concentrated on adding chemical groups like nitro and halogen components to the molecular structure in order to raise the values of the AATSC8s and VE3_Dzs descriptors, and avoiding the addition of hydroxy groups, which were discovered to have adverse effects on the intended activity. We were also able to lower the values of nHsOH, CIC1, and RotBFrac. As indicated in Table 3, seven interesting novel compounds were created by applying these recommended structural alterations to the triazole derivatives. Comparing these compounds to the most active one in the series (Table 4), each one showed a higher pIC_50_ percentage.

### 2.3. Applicability Domain

The obtained leverage values (hi = xit × (Xt × X) − 1 × xi (i = 1, 2, 3... n)) for the designed molecules were compared with the warning leverage (h*). The compound was recommended to be inside the applicability domain of the model based on the leverage (hi) being smaller than the warning leverage (h*). In this case, the superscript t denotes the transpose of the matrix or vector of the designed molecules, n is the number of training set compounds, and k is the number of model descriptors. xi is the matrix of model descriptors of each designed molecule, and X is the matrix of model descriptor values for n training set compounds [14]. Using the leverage threshold h* as a guide, we computed the leverages of every molecule proposed. Based on the values of hi, which has the range of (0.055–0.358), the findings displayed in Table 4 indicate that all of these compounds are acceptable.

### 2.4. Molecular Docking

**Figure 2:**
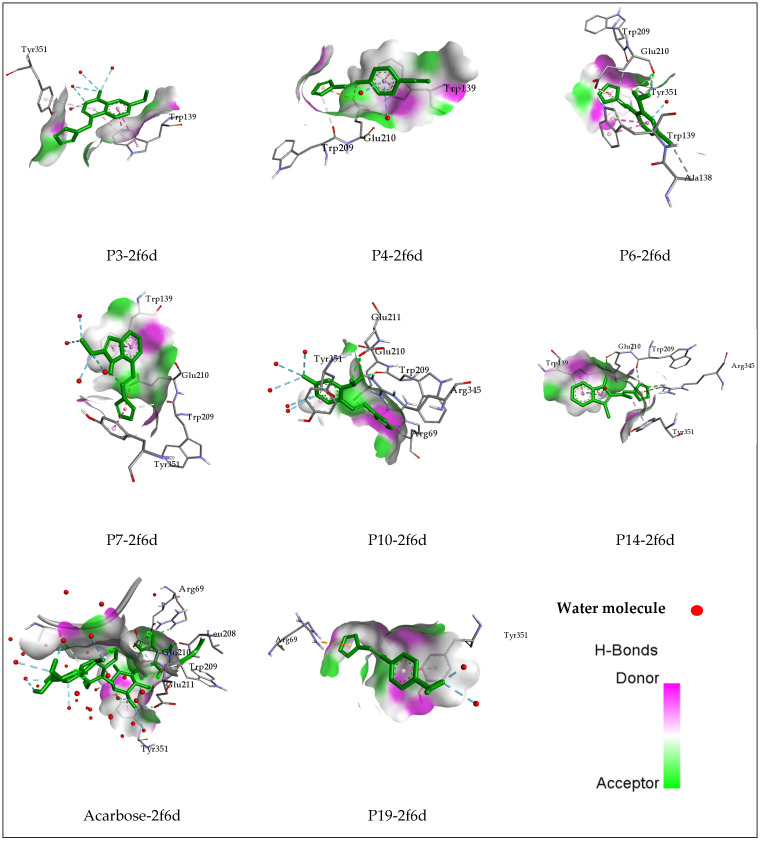
Three-dimensional visualization of the docked ligands into the binding site of alpha-glucosidase receptor.

**Figure 3:**
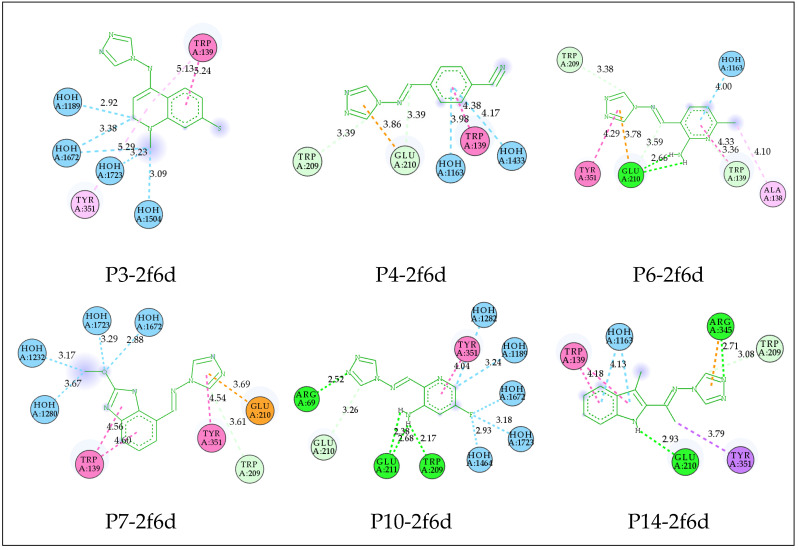

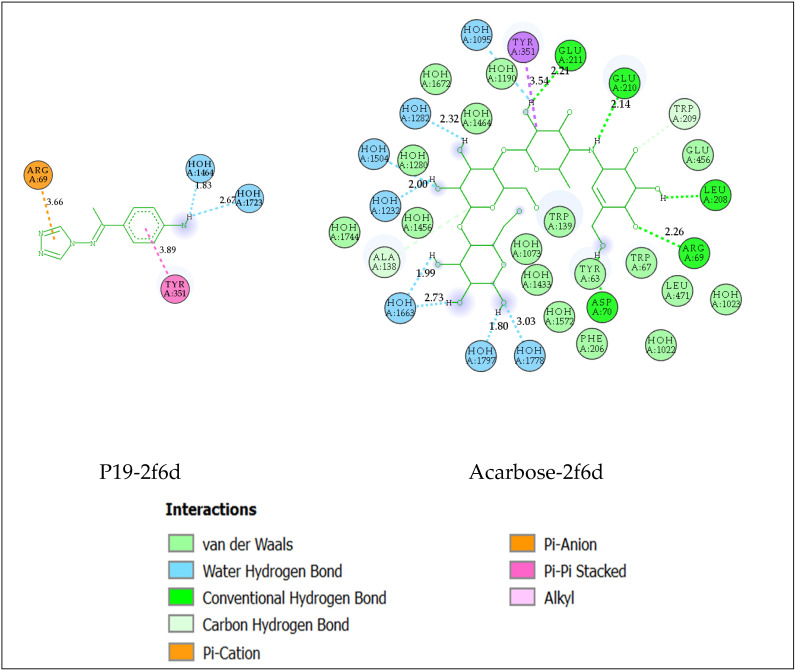
The 2D visualization of created complexes after molecular docking, with distances, interaction types, and participating residues.

The molecular docking of all investigated compounds was conducted within the active site of the target receptor. The findings are illustrated in Figure 2 and Figure 3 and Table 5 and demonstrate favorable binding affinities for all complexes, attributed to the diverse interactions established between the ligands and key residues situated in the binding site. Acarbose, recognized as an alpha-glucosidase inhibitor, was included in the docking simulations to elucidate the interactions formed within the receptor. The Acarbose-2f6d complex revealed notable interactions, including five Conventional Hydrogen Bonds with Arg69, Glu211, Glu210, Leu208, and Asp70 residues, as well as two Carbon–Hydrogen Bonds with Trp209 and Ala138, and a Pi–Sigma interaction with Tyr351. Distances for these interactions ranged between 1.84 Å and 3.79 Å. Moreover, Water Hydrogen bonds were detected, underscoring the involvement of Water molecules in the formation of this intricate complex.

These interactions observed, including Conventional Hydrogen Bonds, Carbon–Hydrogen Bonds, and Pi–Sigma interaction with key residues and additional interactions with Water molecules, are indicative of a complex and intricate binding pattern within the active site of the receptor. Such interactions are crucial and may play a pivotal role in inhibiting the activity of the alpha-glucosidase enzyme. The binding affinity and specificity of the ligands towards the receptor, as evidenced by these interactions, suggest a potential mechanism for effective inhibition, contributing to the therapeutic impact of the studied compounds on alpha-glucosidase activity.

Compound P3 engaged in intricate interactions within the receptor’s active site, including two Pi–Pi Stacked and two Pi–Alkyl interactions with Trp139 and Tyr351 residues. Additionally, Water Hydrogen Bonds were observed with HOH1163, HOH1189, HOH1672, HOH1504, HOH1672, and HOH1723 at distances ranging between 2.92 and 5.29 Å.

For Compound P4, a complex was formed through a Carbon–Hydrogen Bond, Pi–Anion, and Pi–Pi Stacked interactions with Trp209, Glu210, and Trp139 residues. Water Hydrogen Bonds with HOH1433 were also detected.

The complex formed by Compound P6 involved a spectrum of interactions, including a Conventional Hydrogen Bond, Carbon–Hydrogen Bond, Pi–Anion, Pi–Pi Stacked, and Alkyl interactions with Trp139, Trp209, Glu210, Tyr351, and Ala138. A Water Hydrogen Bond with HOH1163 was also identified. Further, Compound P7 created a complex through a Carbon–Hydrogen Bond, Pi–Anion, and Pi–Pi Stacked interactions with Trp209, Glu210, Tyr351, Trp139, and Water Hydrogen Bonds with HOH1282, HOH1672, HOH1723, HOH1232, and HOH1280.

In the case of Compound P10, a complex was formed through a Conventional Hydrogen Bond, Carbon–Hydrogen Bond, and Pi–Pi Stacked interactions with Arg69, Trp209, Glu211, Glu210, and Tyr351. Water Hydrogen Bonds were observed with HOH1464, HOH1672, HOH1723, HOH1189, and HOH1282.

Compound P14 exhibited a diverse array of interactions, including a Conventional Hydrogen Bond, Carbon–Hydrogen Bond, Pi–Cation, Pi–Sigma, and Pi–Pi Stacked interactions with Arg345, Glu210, Trp209, Tyr351, and Trp139 residues, while the complex formed by Compound 19 involved Pi–Cation and Pi–Pi Stacked interactions with Arg69 and Tyr351, along with Water Hydrogen Bonds with HOH1723 and HOH1464.

Among the compounds subjected to docking, ligands P6, P10, and P14 were anticipated to establish interactions similar to Acarbose, including Conventional Hydrogen bonds, Carbon–Hydrogen bonds, and Pi–Sigma interactions. These interactions were projected to involve the same residues, such as Arg69, Trp209, Glu210, Glu211, and Tyr351. This anticipation suggests a potential similarity in the binding mechanism between these ligands and the reference drug, indicating a likelihood of shared effects within the studied receptor. This observation implies that these ligands may operate through a comparable mechanism to Acarbose, demonstrating a potential therapeutic impact on the target receptor.

### 2.5. ADMET Properties Prediction

The pharmacokinetic properties of the studied compounds were predicted using the pkCSM online tools. The results of the analysis are listed in Table 6. It was found that all investigated compounds adhere to the Lipinski rules.

All of the substances are expected to have high absorption from the gastrointestinal (GI) tract into the bloodstream, indicating their potency for absorption, based on the information provided in Table 6. Additionally, the compounds showed excellent Water solubility, which is advantageous for absorption. All compounds except for Compounds P10 and P19 exhibited strong Caco-2 permeability.

Since none of the substances were expected to be P-glycoprotein inhibitors, it is unlikely that they will obstruct the efflux transporters that allow medications to be pumped out of cells. Compounds P7 and P14, on the other hand, were predicted to be P-glycoprotein substrates, which means P-glycoprotein could be able to detect and transport them. Furthermore, it was noted that all compounds had a high level of skin permeability, signifying its capacity to penetrate the epidermal barrier and enter the body. Here, a LogKp value of less than −2.5 is considered high-skin-permeability.

All compounds were predicted to have a low volume of distribution at steady state (VDss) values, except Compounds P3 and P7. In this context, a low VDss value is defined as a LogVDss value less than −0.15, indicating that in a steady state, the chemicals have a relatively small volume of distribution throughout the body. Compound P14 was predicted to have a modest potential to cross the blood–brain barrier, as demonstrated by a LogBB (logarithm of the blood–brain barrier partition coefficient) higher than 0.3. The remaining compounds were estimated to have a modest potential to cross. Compound 3 was found to have the potency to penetrate the central nervous system (CNS), while Compounds P6, P7, and P10 were considered unable to penetrate the CNS.

There was a low chance that any of the substances would interact with any of these specific cytochrome P450 enzymes because it was anticipated that they would all be inhibitors of CYP1A2 and neither substrates nor inhibitors of CYP2D6, CYP2C19, CYP2C9, or CYP3A4. Nevertheless, it was considered that Compounds P6 and P14 were CYP3A4 substrates, indicating that the CYP3A4 enzyme could metabolize them.

Except for Compound P7, which was thought to be an OCT2 substrate and may be carried via the kidney’s OCT2 transporter, other compounds were predicted to be non-renal OCT2 substrates. The range of the medications’ total clearance values (Log(ml/min/kg)) indicates potential dose rates required to achieve steady-state concentrations, from 0.063 to 0.586.

Since the compounds were projected to be neither hERG I nor hERG II inhibitors, it is unlikely that they will have a major impact on the hERG channel, which is crucial for heart function. Additionally, it was anticipated that they would not produce skin sensitization, which means that when they come into contact with skin, they will likely not trigger allergic responses.

According to the AMES test, chemicals P6, P10, and P14 were expected to not introduce AMES toxicity, indicating that it is unlikely that they will result in bacterial cell mutations.

### 2.6. Molecular Dynamics Simulation

Ligands P6, P10, and P14 were selected for molecular dynamics simulation based on their favorable pharmacological properties, good binding scores, and interactions with key residues of the analyzed enzyme.

#### 2.6.1. Root-Mean-Squared Deviation

**Figure 4:**
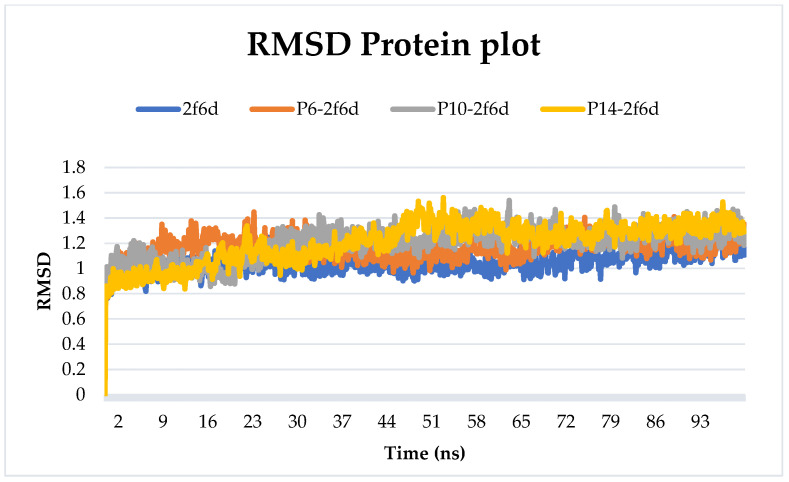
Protein RMSD plot of all simulated complexes after 100 ns.

A 100 ns simulation was conducted on the protein–ligand complexes (2f6d with P6, P10, and P14) and the uncomplexed protein to observe any deviations or structural changes induced when the protein was bound to the selected ligands. The Root-Mean-Square Deviation (RMSD) values for the proteins were computed and are visualized in Figure 4. The outcomes revealed a consistent state with an RMSD consistently below 3 Å over the entire simulation period. This signifies that the complexes attained a stable conformation, indicating stability despite their interactions with the examined ligands. The persistent and low RMSD values suggest that the complexes maintained structural integrity throughout the simulation.

**Figure 5:**
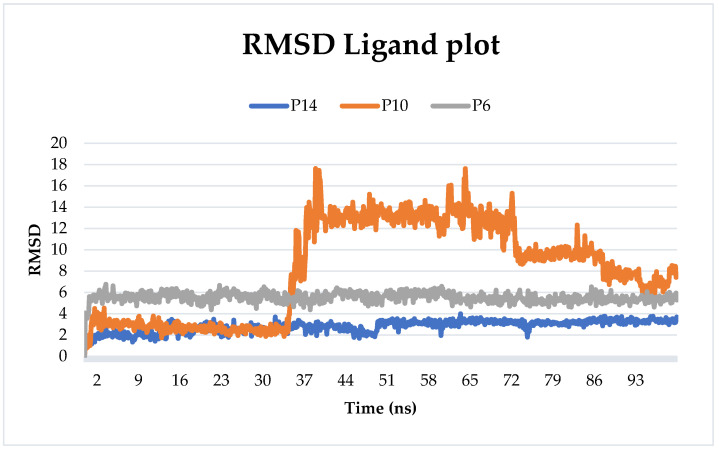
Ligand RMSD plot of all simulated complexes after 100 ns.

Figure 5 illustrates the RMSD values of the proposed ligands during their interaction with the target. The findings suggest stability throughout the simulation for P6 and P14, with average RMSD values of 5.52 Å and 2.82 Å, respectively. In contrast, P10 initially maintained a consistent state for the first thirty nanoseconds before exhibiting fluctuations exceeding 15 Å, and then a decrease in RMSD value was eventually noted towards the end of the simulation. The average RMSD of P10 was 8.16 Å.

#### 2.6.2. Root-Mean-Squared Fluctuation

**Figure 6:**
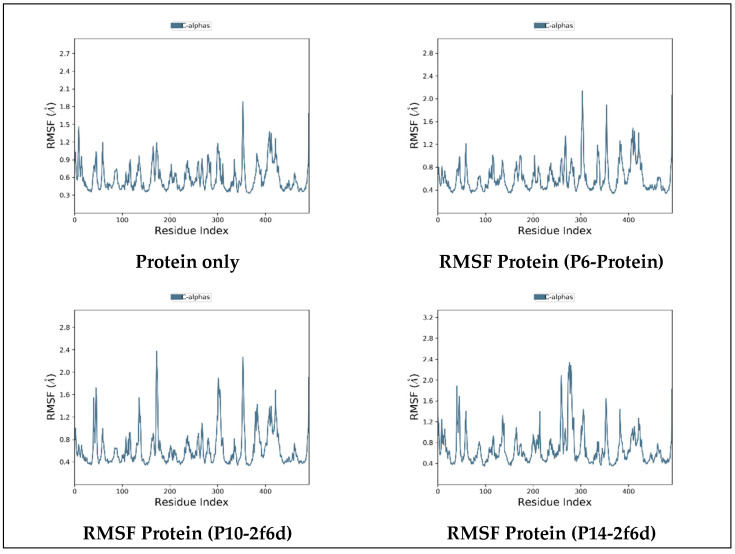
Protein RMSF plot of all simulated complexes after 100ns.

The investigation included a Root-Mean-Squared Fluctuation (RMSF) analysis for all simulated complexes, and a separate simulation was conducted for the uncomplexed protein to compare residue fluctuations during the simulation period. The primary objective of this analysis was to evaluate the stability of protein residues in the presence of the investigated ligands. The RMSF values for protein residues remained below 3 Å for all simulated complexes (Figure 6), indicating the absence of significant fluctuations. This implies that a state of stability was achieved for the protein residues despite their interactions with the studied ligands.

#### 2.6.3. Protein–Ligand Contact

The ligand–protein interactions were thoroughly investigated, uncovering a diverse range of molecular bonds and bridges contributing to their binding affinity, as illustrated in Table 7. Specifically, P6 formed Hydrogen bonds with Tyr63, indicating a directed interaction. Hydrophobic interactions played a crucial role, with P6 engaging hydrophobic residues Trp209, Tyr351, Trp362, and Trp473, enhancing complex stability. Ionic bonds with Trp67, Asp70, and Glu210 added an electrostatic dimension to the interaction. Water molecules acted as bridges, mediating specific interactions with Tyr63, Arg69, Asp70, Trp209, and Glu210. The persistence of interactions, particularly with Asp63, Asp70, Trp209, Glu210, Tyr351, and Trp362 residues, underscored their crucial role in maintaining complex stability.

For P10-2f6d and P14-2f6d, the ligand–protein interactions revealed a nuanced network of molecular bonds contributing to their binding affinity. P10 engaged in diverse and specific interactions, forming Hydrogen bonds with Trp67, Gly140, Trp209, Glu210, Glu211, and Arg345. Water bridges with Arg69-, Asp207-, Leu208-, Trp209-, Glu210-, Glu211-, and Arg345-mediated specific contacts. Hydrophobic bonds with Lys127, Trp139, Phe206, and Tyr351 enhanced complex stability, while ionic bonds with Glu210 and Tyr351 introduced an electrostatic dimension. P14 formed ionic bonds with Tyr63 and Glu210, indicating specific electrostatic interactions. Hydrophobic bonds with Tyr63, Trp209, Tyr351, and Trp62 contributed to complex stability. Water bridges involving Ala54, Arg69, Asp70, Leu208, Glu210, and Trp473 facilitated specific contacts. Hydrogen bonds with Tyr63 and Trp209 enriched the interaction profile, providing comprehensive insights into the molecular basis of their binding mechanisms. Persistent interactions were consistently observed throughout the simulation, establishing connections between P14 and residues Tyr63, Asp70, Trp209, Glu210, and Tyr351, suggesting the role of these interactions in maintaining the stability of the formed complex P14-2f6d.

#### 2.6.4. Binding Free Energy

The binding free energy calculations for the three simulated complexes, P6-2f6d, P10-2f6d, and P14-2f6d, revealed favorable thermodynamic stability. The negative values of ΔG (−32.59 kcal/mol, −35.8 kcal/mol, and −41.17 kcal/mol, respectively) indicate spontaneous and energetically favorable interactions between the ligands and the protein. These results suggest strong binding affinities, with P14-2f6d exhibiting the highest negative ΔG, highlighting its potentially robust and stable interaction with the target protein.

## 3. Material and Methods

A database containing 33 substituted 1,2,4-triazoles was retrieved from a previous study [14]. These compounds exhibited moderate-to-high activity against α-glucosidase, as listed in Table 8, where their IC_50_ values were transformed into pIC_50_ values (-logIC_50_) for QSAR modeling. The structures of the studied compounds were generated and optimized under the MMFF94 force field by using Chem3D V.19.0.0.22 [22].

### 3.1. Molecular Descriptor Computation and Pruning

The PaDEL tool was utilized to calculate the molecular descriptors of the substances [23]. PaDEL offers over 800 molecular characteristics for every molecule; hence, it was required to filter these descriptors to only contain pertinent data. For this, the objective feature selection module from QSARINS software [24] was employed. Descriptors with high co-linearity (|r| > 0.90) and virtually constant (>95%) values were excluded to avoid multi-collinear and spurious variables in the GA-MLR models. A dataset including 433 molecular descriptors encompassing mono-dimensional (1D-) and bi-dimensional (2D-) descriptor spaces was retained after the descriptor reduction step [25,26].

### 3.2. QSAR Model Construction and Validation

The QSARINS software was utilized to build the QSAR models. This program is well known for producing statistically strong GA-MLR-based QSAR models. QSARINS’s random splitting option was used to divide the dataset into training and test sets at random for this purpose [27]. Twenty percent of the compounds were in the test set and eighty percent were in the training set [28]. The models were generated using a representative subset of the dataset and assessed on a different collection of substances thanks to this tried-and-true methodology [29].

By including the maximum number of molecular descriptors impacting or altering biological potential/activity, this method aimed to enhance the performance of the models [30]. The calculated molecular descriptors are used as independent variables to predict or explain the activity of a molecule by employing Formula (1):

Y = a_0_ + a_1_X_1_ + a_2_X_2_ (1)

In accordance with the OECD guidelines [31,32], the obtained models underwent comprehensive external and internal statistical validations, Y-randomization, and applicability domain analysis. A variety of model evaluation metrics and statistical parameters were taken into account to assess the models’ performance and choose the best model. These included the coefficient adjusted for degrees of freedom (R^2^_adj_), coefficient of determination for the test set (R^2^_test_), root-mean-squared error (RMSE), and R-squared coefficient of determination (R^2^). As the fitness function, the leave-one-out cross-validation coefficient, or Q^2^_LOO_, was also given. Typically, measurements with values higher than 0.6 signify a more stable and consistent model [33,34,35].

Leverage analysis—expressed as William’s plot—is used in chemometrics and QSAR analysis to assess a model’s applicability domain (AD) by determining the standardized residuals (r) and the leverage threshold values (h* = (3 × (k + 1))/n), where n denotes the number of trainings and k the number of descriptors. The range that the model is thought to be dependable for forecasting new values is represented by the AD [36,37].

### 3.3. Molecular Docking

The molecular docking method is widely used to predict the best poses of the examined ligands when docked in the active pocket of the target, as well as the binding affinity values and the created molecular interactions between the residues located in the receptor and the studied ligands [38].

The receptor utilized in this investigation was sourced from the Protein Data Bank, identified by the PDB ID of 2f6d [39], corresponding to the complex structure involving a glucoamylase from Saccharomycopsis fibuligera and Acarbose.

**Figure 7:**
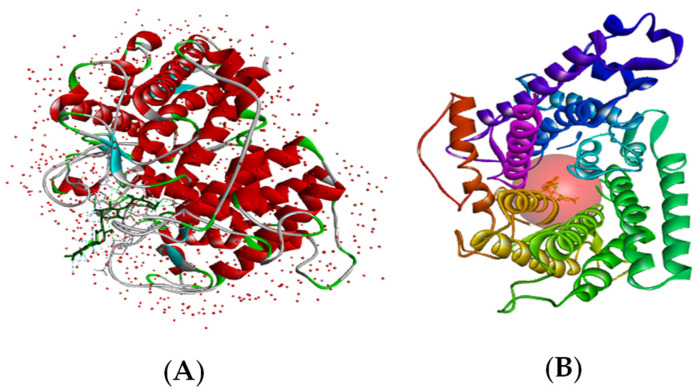
(**A**) The 3D visualization of the 2f6d receptor; (**B**) the binding site of the 2f6d receptor.

The selection of this particular structure was influenced by its intricate nature, resembling that of Acarbose. Acarbose is renowned for its effectiveness in inhibiting the alpha-glucosidase enzyme. Furthermore, the high resolution of this structure, standing at 1.60 Å (below 2 Å), adds to its suitability. The receptor comprises 492 amino acids in the ‘Chain A’, referred to as glucoamylase (Figure 7A). It also contains additional heteroatoms such as alpha-acarbose, phosphate, and sodium ions, which were excluded before the commencement of the molecular docking phase [39].

In preparation for the docking simulations, the receptor underwent necessary adjustments, including minimizing the energy of the protein structure using Swiss PDB Viewer [40], the addition of polar Hydrogens, and computation of Gasteiger charges using AutoDock vina software [41,42,43]. In this case, given the presence of Water molecules within the binding site of the protein, they were preserved to explore potential interactions with the docked ligands. The ligands underwent an energy minimization process employing the MMFF94 force field through Avogadro software [44]. This step was undertaken to refine and optimize the structural conformation of the ligands.

The choice of the grid box for docking was strategic, determined by the initial position of the co-crystalized ligand (Acarbose), which is acknowledged as an inhibitor of alpha-glucosidase activity [45], serving as the reference drug for comparative analysis of interactions. The coordinates of the binding site were meticulously set at x = 12.68 Å, y = 10.80 Å, z = −6.35 Å, with a size of 20 Å^3^ and a space center of 0.375 Å (Figure 7B).

**Figure 8:**
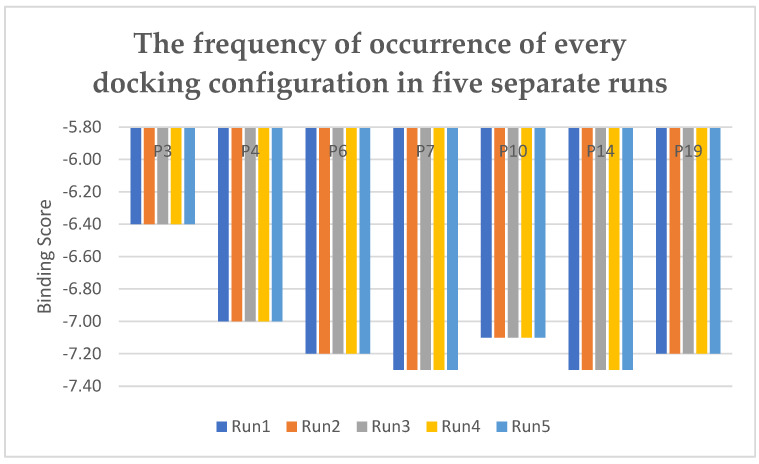
Frequency of appearance for each docking conformation in five independent runs.

The molecular docking process was iterated five times to ensure robustness and reliability. The conformations of the ligands were selected based on their frequency of appearance across these multiple runs. Notably, all chosen conformations exhibited consistent presence in every run, affirming their reliability and reinforcing the confidence in their representativeness. Figure 8 provides a visualization of the frequency for the obtained conformations in each run.

**Figure 9:**
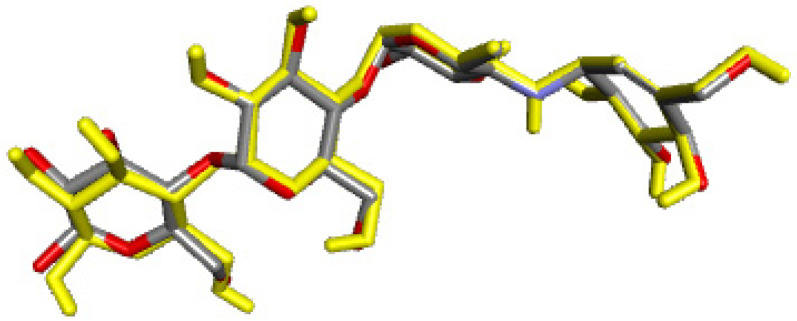
The alignment of the docked ligand (Yellow) with the initial ligand (Grey) illustrates a noteworthy similarity, affirming the reliability of the molecular docking protocol.

The co-crystalized ligand (Acarbose) was initially docked into the binding site of the receptor to validate the docking protocol [8]. Subsequently, AutoDock tools [46] facilitated the molecular docking of all designed compounds, enabling an in-depth exploration of potential interactions and the determination of binding affinities within the active site of the receptor. The docking simulation was conducted through a total of 9 runs, and the complex resulting from the run with the lowest binding affinity, corresponding to an RMSD of 0, was selected and subjected to analysis [47]. The calculated Root-Mean-Squared Deviation (RMSD) value of 0.217 Å (below 2 Å) indicates a minimal deviation between the initial ligand and the docked ligand (Figure 9). This result affirms the precision of the docking protocol in faithfully reproducing the binding pose of the reference ligand within the active site of the receptor [48].

### 3.4. ADMET Analysis

The designed compounds with a high value of pIC_50_ against alpha-glucosidase activity were subjected to an ADMET analysis for the purpose of gaining insight into the pharmacological properties which includes several properties such as the absorption, distribution, metabolism, excretion, and toxicity [49,50]. The pkCSM website is widely used for predicting these properties and evaluating the behavior of compounds in the human body.

Lipinski’s rules were used to eliminate the compounds that do not respect the threshold values of these principles, including a molecular weight of less than 500 g/Mol, no more than 5 donor bonds, no more than 10 acceptor bonds, and a partition coefficient (LogP) no more than 5 [51]. Additionally, several properties were evaluated to determine the behavior of the analyzed compounds in the human body [51] such as Caco-2 permeability, Intestinal absorption (human), and the volume of distribution at steady state (VDss). These evaluations offer interesting details into the potential behavior and suitability of the compounds for further development.

### 3.5. Molecular Dynamics (MD) Simulation

To gain insight into the structural changes that befell the protein and ligand through key parameters like the (RMSD) Root-Mean-Squared Deviation and (RMSF) Root-Mean-Squared Fluctuation, the hit compounds with the best binding score, highest biological activity, and good pharmacological properties were put through a molecular dynamics’ simulation. The generated molecular interactions between them were then assessed to determine the cause of stability or changes observed in the protein and ligand structures [52,53].

The generated complexes were prepared, minimized, and optimized under the OPLS3e force field [54] using the protein preparation wizard which is available in Maestro software [55]. More recently, we used the Water model (TIP3P) to build an orthorhombic simulation [56]. Following the addition of Na^+^ and Cl^−^ counterions to neutralize the charge of the solvated systems, the physiological salt concentration was adjusted to 0.15 M. After that, the system was heated gradually to the target temperature (which is under 300 K and one bar of pressure) using the Nose–Hoover thermal algorithm and the Martina–Tobias-Klein method [57]. A recording interval, an isothermal–isobaric ensemble (NPT), and an MD simulation duration of 100 ns were all employed. In this work, MD simulations were performed using the Desmond package [58], which is part of the Schrödinger 2020-3 academic program.

The Molecular Mechanics/Generalized Born Surface Area (MM/GBSA) technique, a crucial component of the Maestro software package, was used to calculate the binding free energy for the simulated complexes. The binding free energies in molecular dynamics simulations were estimated using MM/GBSA. It is especially helpful for comprehending the thermodynamics of ligand binding in molecular dynamics simulations and offers a thorough and effective method of analyzing the energetics of protein–ligand interactions [59].

## 4. Conclusions

This study employed a comprehensive QSAR analysis of 33 triazole derivatives to identify the key factors influencing their inhibitory activity against alpha-glucosidase. This analysis aimed to identify novel potential compounds that could serve as novel medications for diabetes mellitus. Based on the best selected MLR model, seven promising compounds were designed as potential drugs, and subjected to the molecular docking and dynamics simulations to gain insights into the stability and the mode of interaction between the designed compounds and the target enzyme. The pharmacokinetic properties of the compounds were assessed to predict their behavior in the human body through various parameters such as absorption, distribution, metabolism, and excretion of the compounds. The results highlighted three novel compounds (P6, P10, and P14) with high biological activity, strong binding affinity to the target enzyme, favorable thermodynamic properties, and suitability for oral administration.

## Figures and Tables

**Table 1:** The developed model using GA-MLR and its evaluated statistical parameters.

Model Equation					
pIC_50_ = 6.403 + 0.759 AATSC8s + 0.022 VE3_Dzs − 0.112 nHsOH − 0.338 CIC1 − 1.804 RotBFrac					
Set	33	Training set	27	Test set	6
Fitting criteria		Internal validation criteria		External validation criteria	
R^2^	0.767	Q^2^_LOO_	0.649	R^2^_ext_	0.633
R^2^_adj_	0.712	Random Models Parameters			
R^2^-R^2^_adj_	0.055	Average R	Average R^2^	Average Q^2^	cRp^2^
RMSE	0.082	0.428	0.196	−0.342	0.669

**Table 2:** The significance of the molecular descriptors constructed the developed model.

Descriptor Symbol	Name of Descriptor	References
AATSC8s	Averaged and centered Moreau–Broto autocorrelation of lag 8 weighted by intrinsic state	[17]
VE3_Dzs	Logarithmic coefficient sum of the last eigenvector from Barysz matrix weighted by Sanderson EN	[18]
nHsOH	The count of a specific atom-type Hydrogen (H) E-State associated with hydroxy groups (OH)	[19]
CIC1	1-ordered complementary information content	[20]
RotBFrac	Fraction of rotatable bonds, excluding terminal bonds	[21]

**Table 3:** The 2D visualization of the new designed compounds using the developed model.

N	Structure	N	Structure
3	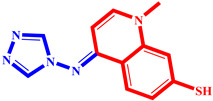	10	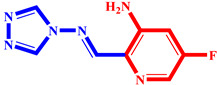
4	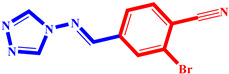	14	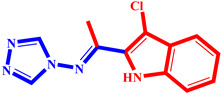
6	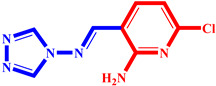	19	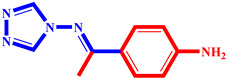
7	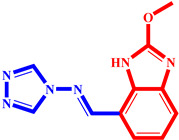		

**Table 4:** The values of the descriptors constructing the developed model for the synthetized compounds, designed compounds with their calculated pIC_50_, and leverages.

Name	N°	AATSC8s	VE3_Dzs	nHsOH	CIC1	RotBFrac	pIC_50_	hi
Synthetized Compounds	1 *	−0.006	−3.819	1	1.439	0.133	5.582	h* = 0.667
	2 *	−0.010	−6.599	2	1.316	0.125	5.264	
	3	0.037	−7.797	2	1.316	0.125	5.264	
	4	−0.010	−8.587	2	1.316	0.125	5.270	
	5	−0.075	−5.287	3	1.328	0.118	5.241	
	6	0.064	−7.498	0	1.578	0.222	5.369	
	7 *	0.043	−6.004	0	1.578	0.222	5.367	
	8 *	0.184	−4.740	0	1.830	0.250	5.263	
	9	0.131	−5.434	0	1.633	0.263	5.271	
	10	0.122	−7.007	0	1.633	0.263	5.262	
	11	−0.174	−3.355	0	1.621	0.125	5.369	
	12 *	−0.169	−1.724	0	1.968	0.167	5.196	
	13	−0.128	−2.264	0	1.507	0.133	5.368	
	14	−0.128	−2.531	0	1.346	0.125	5.604	
	15	−0.127	−7.233	0	1.376	0.177	5.270	
	16	0.044	−4.375	0	1.376	0.177	5.607	
	17	−0.074	−2.618	1	2.214	0.182	5.198	
	18 *	0.032	−7.781	1	1.193	0.176	5.276	
	19	0.256	−4.965	1	1.396	0.211	5.485	
	20	0.220	−5.270	0	1.349	0.211	5.599	
	21	−0.004	−2.950	1	1.103	0.125	5.625	
	22	−0.021	−6.069	1	0.937	0.167	5.599	
	23	−0.103	−3.622	0	1.161	0.176	5.461	
	24	0.012	−5.284	0	1.161	0.176	5.633	
	25	−0.010	−6.839	0	1.161	0.176	5.644	
	26	−0.127	−9.690	0	1.055	0.167	5.359	
	27	−0.030	−6.929	1	1.011	0.167	5.636	
	28	−0.080	−4.901	0	1.069	0.133	5.613	
	29	0.001	−3.755	0	2.216	0.217	5.264	
	30	−0.037	−3.692	0	2.094	0.240	5.184	
	31	0.000	−4.566	0	2.641	0.083	5.267	
	32	0.026	−4.196	0	2.720	0.074	5.201	
	33	−0.019	−7.010	0	1.664	0.111	5.680	
Designed compounds	P3	−0.106	−3.082	0	1.143	0.050	5.780	0.084
	P4	−0.005	−3.524	0	1.103	0.118	5.738	0.023
	P6	−0.084	−3.328	0	0.891	0.125	5.741	0.059
	P7	0.083	−4.032	0	1.036	0.150	5.758	0.055
	P10	0.077	−5.263	0	0.800	0.125	5.852	0.082
	P14	0.113	−8.222	0	1.224	0.100	5.717	0.358
	P19	0.109	−3.410	0	1.318	0.125	5.741	0.081

*: test compounds, P: proposed compounds.

**Table 5:** The created interactions of obtained complexes with binding scores, participated residues, molecular interactions, and distances expressed in Å.

N	Score Kcal/Mol	Residue	Interaction Type	Distance (Å)
P3	−6.4	Trp139	Pi–Pi Stacked	4.87
		Trp139	Pi–Pi Stacked	4.19
		Trp139	Pi–Alkyl	5.13
		Tyr351	Pi–Alkyl	5.29
		HOH1163	Water Hydrogen Bond	3.57
		HOH1189	Water Hydrogen Bond	2.92
		HOH1672	Water Hydrogen Bond	3.38
		HOH1504	Water Hydrogen Bond	3.09
		HOH1672	Water Hydrogen Bond	3.45
		HOH1723	Water Hydrogen Bond	3.23
P4	−7	Trp209	Carbon–Hydrogen Bond	3.39
		Glu210	Carbon–Hydrogen Bond	3.39
		Glu210	Pi–Anion	3.85
		Trp139	Pi–Pi Stacked	3.85
		Trp139	Pi–Pi Stacked	4.36
		HOH1433	Water Hydrogen Bond	4.17
P6	−7.2	Glu210	Conventional Hydrogen Bond	2.62
		Glu210	Conventional Hydrogen Bond	2.66
		Trp139	Carbon–Hydrogen Bond	3.36
		Trp209	Carbon–Hydrogen Bond	3.38
		Glu210	Carbon–Hydrogen Bond	3.59
		Glu210	Pi–Anion	3.77
		Trp139	Pi–Pi Stacked	4.59
		Trp139	Pi–Pi Stacked	3.97
		Tyr351	Pi–Pi Stacked	4.29
		Ala138	Alkyl	4.1
		HOH1163	Water Hydrogen Bond	4
P7	−7.3	Trp209	Carbon–Hydrogen Bond	3.61
		Glu210	Pi–Anion	3.69
		Trp139	Pi–Pi Stacked	4.43
		Trp139	Pi–Pi Stacked	3.74
		Tyr351	Pi–Pi Stacked	4.54
		Trp139	Pi–Pi Stacked	4.56
		HOH1282	Water Hydrogen Bond	3.81
		HOH1672	Water Hydrogen Bond	2.88
		HOH1723	Water Hydrogen Bond	3.29
		HOH1232	Water Hydrogen Bond	3.17
		HOH1280	Water Hydrogen Bond	3.67
P10	−7.1	Arg69	Conventional Hydrogen Bond	2.52
		Trp209	Conventional Hydrogen Bond	2.17
		Glu211	Conventional Hydrogen Bond	2.68
		Glu211	Conventional Hydrogen Bond	2.38
		Glu210	Carbon–Hydrogen Bond	3.26
		Tyr351	Pi–Pi Stacked	4.04
		HOH1464	Water Hydrogen Bond	2.93
		HOH1672	Water Hydrogen Bond	3.48
		HOH1723	Water Hydrogen Bond	3.18
		HOH1189	Water Hydrogen Bond	3.24
		HOH1282	Water Hydrogen Bond	3.39
P14	−7.3	Arg345	Conventional Hydrogen Bond	2.71
		Glu210	Conventional Hydrogen Bond	2.93
		Trp209	Carbon–Hydrogen Bond	3.08
		Arg345	Pi–Cation	3.42
		Tyr351	Pi–Sigma	3.79
		Trp139	Pi–Pi Stacked	5.27
		Trp139	Pi–Pi Stacked	4.73
		Trp139	Pi–Pi Stacked	3.97
		Trp139	Pi–Pi Stacked	4.45
P19	−7.2	Arg69	Pi–Cation	3.66
		Tyr351	Pi–Pi Stacked	3.89
		HOH1723	Water Hydrogen Bond	2.67
		HOH1464	Water Hydrogen Bond	1.83
Acarbose	−12.3	Arg69	Conventional Hydrogen Bond	2.25
		Glu211	Conventional Hydrogen Bond	2.21
		Glu210	Conventional Hydrogen Bond	2.14
		Leu208	Conventional Hydrogen Bond	1.9
		Asp70	Conventional Hydrogen Bond	1.84
		Trp209	Carbon–Hydrogen Bond	3.52
		Ala138	Carbon–Hydrogen Bond	3.79
		Tyr351	Pi–Sigma	3.54
		HOH1449	Water Hydrogen Bond	2.72
		HOH1504	Water Hydrogen Bond	2.91
		HOH1778	Water Hydrogen Bond	3.03
		HOH1797	Water Hydrogen Bond	1.8
		HOH1663	Water Hydrogen Bond	2.73
		HOH1663	Water Hydrogen Bond	1.99
		HOH1232	Water Hydrogen Bond	2
		HOH1282	Water Hydrogen Bond	2.32
		HOH1095	Water Hydrogen Bond	3.01

**Table 6:** The predicted ADMET properties of analyzed compounds by using pkCSM online tools.

Compounds	P3	P4	P6	P7	P10	P14	P19
MOL_WEIGHT	257.322	276.097	222.639	242.242	206.184	259.7	201.233
LOGP	1.4225	1.79448	0.7909	1.0452	0.2766	2.6851	1.1326
ROTATABLE_BONDS	1	2	2	3	2	2	2
ACCEPTORS	6	5	6	6	6	4	5
DONORS	1	0	1	1	1	1	1
SURFACE_AREA	108.812	100.171	90.409	102.135	84.271	108.105	87.251
Water solubility	−3.072	−3.119	−2.684	−2.956	−2.446	−3.667	−1.893
Caco2 permeability	1.292	1.011	1.294	1.322	0.74	1.336	0.728
Intestinal absorption (human)	98.134	97.517	84.358	77.072	85.844	93.42	72.544
Skin Permeability	−2.618	−2.554	−2.84	−2.735	−2.769	−2.72	−2.897
P-glycoprotein substrate	No	No	No	Yes	No	Yes	No
P-glycoprotein I inhibitor	No	No	No	No	No	No	No
P-glycoprotein II inhibitor	No	No	No	No	No	No	No
VDss (human)	−0.294	−0.351	−0.714	0.051	−0.767	−0.195	−0.367
Fraction unbound (human)	0.339	0.293	0.448	0.378	0.448	0.215	0.311
BBB permeability	−0.043	0.149	−0.764	−0.587	−0.796	0.373	−0.323
CNS permeability	−1.977	−2.814	−3.047	−3.456	−3.118	−2.06	−2.414
CYP2D6 substrate	No	No	No	No	No	No	No
CYP3A4 substrate	Yes	No	No	No	No	Yes	No
CYP1A2 inhibitor	Yes	Yes	Yes	Yes	Yes	Yes	Yes
CYP2C19 inhibitor	No	No	No	No	No	No	No
CYP2C9 inhibitor	No	No	No	No	No	No	No
CYP2D6 inhibitor	No	No	No	No	No	No	No
CYP3A4 inhibitor	No	No	No	No	No	No	No
Total Clearance	0.109	0.063	0.16	0.586	0.557	0.503	0.239
Renal OCT2 substrate	No	No	No	Yes	No	No	No
AMES toxicity	Yes	Yes	No	Yes	No	No	Yes
Max. tolerated dose (human)	−0.749	0.397	0.632	0.593	0.613	−0.25	0.378
hERG I inhibitor	No	No	No	No	No	No	No
hERG II inhibitor	No	No	No	No	No	No	No
Oral Rat Acute Toxicity (LD50)	2.254	2.313	2.379	1.804	2.22	2.51	2.383
Oral Rat Chronic Toxicity (LOAEL)	1.705	1.747	1.604	1.128	0.664	1.669	1.82
Hepatotoxicity	Yes	No	No	Yes	Yes	Yes	No
Skin Sensitization	No	No	No	No	No	No	No
T. Pyriformis toxicity	0.564	1.176	0.284	0.285	0.241	1.002	0.647
Minnow toxicity	2.586	1.524	2.684	2.529	3.021	1.28	2.364

**Table 7:** Protein–ligand contact (histogram and timeline) of all simulated complexes after 100 ns (Pink: ionic bond; bleu: water bridge; violet: hydrophobic bond, and Green: Hydrogen bond).

Complex	PL-Contacts Histogram	PL-Contacts Timeline
P6-2f6d	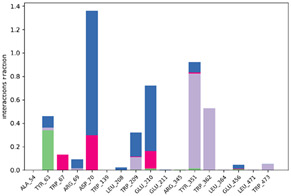	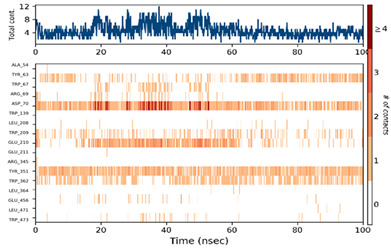
P10-2f6d	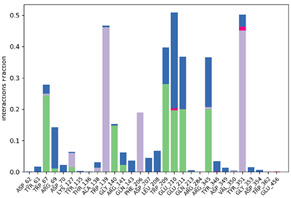	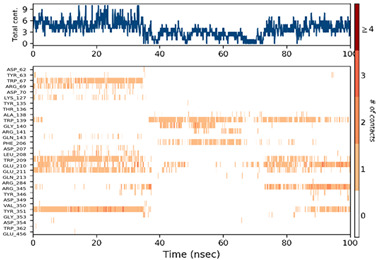
P14-2f6d	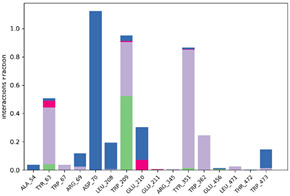	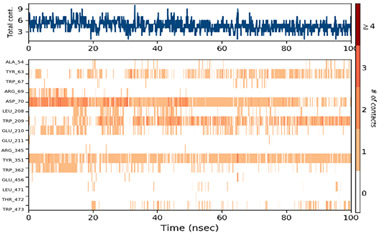

**Table 8:** The 2D structure of 33 triazole derivatives with their biological activity against alpha-glucosidase pIC_50_.

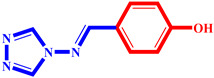 (1) pIC_50_ = 5.58	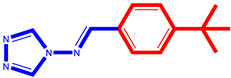 (12) pIC_50_= 5.20	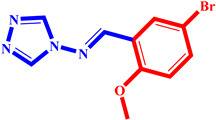 (23) pIC_50_ = 5.46
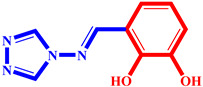 (2) pIC_50_ = 5.26	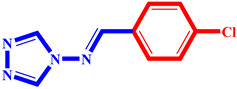 (13) pIC_50_ = 5.37	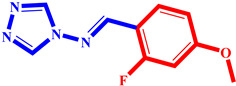 (24) pIC_50_ = 5.63
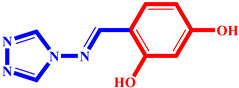 (3) pIC_50_ = 5.26	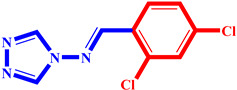 (14) pIC_50_ = 5.60	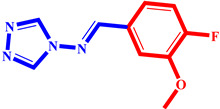 (25) pIC_50_ = 5.64
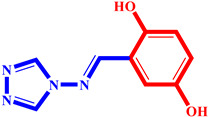 (4) pIC_50_ = 5.27	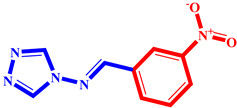 (15) pIC_50_ = 5.27	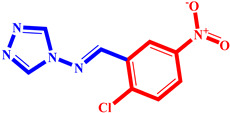 (26) pIC_50_ = 5.36
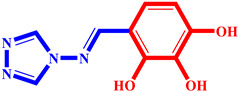 (5) pIC_50_ = 5.24	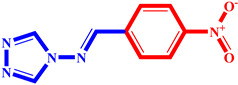 (16) pIC_50_ = 5.61	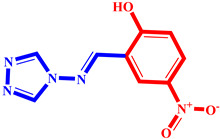 (27) pIC_50_ = 5.64
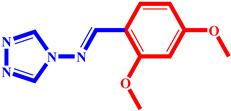 (6) pIC_50_ = 5.37	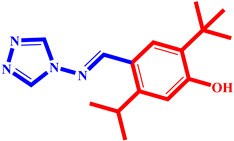 (17) pIC_50_ = 5.20	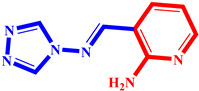 (28) pIC_50_ = 5.61
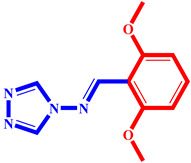 (7) pIC_50_ = 5.37	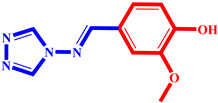 (18) pIC_50_ = 5.28	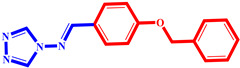 (29) pIC_50_ = 5.26
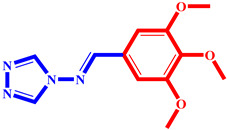 (8) pIC_50_ = 5.26	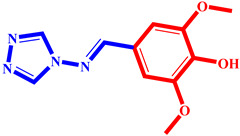 (19) pIC_50_ = 5.48	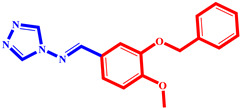 (30) pIC_50_ = 5.18
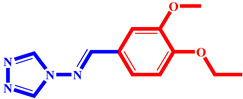 (9) pIC_50_ = 5.27	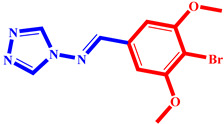 (20) pIC_50_ = 5.60	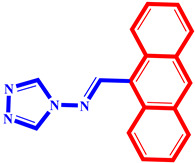 (31) pIC_50_ = 5.27
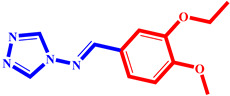 (10) pIC_50_ = 5.26	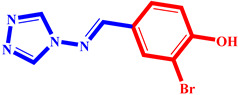 (21) pIC_50_ = 5.62	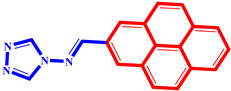 (32) pIC_50_ = 5.20
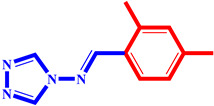 (11) pIC_50_ = 5.37	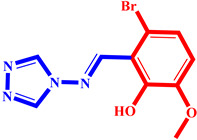 (22) pIC_50_ = 5.60	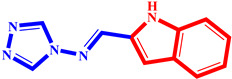 (33) pIC_50_ = 5.68

## Data Availability

Data are contained within the article.

## References

[1] Sarwar N., Gao P., Seshasai S.R., Gobin R., Kaptoge S., Di Angelan-tonio E., Ingelsson E., Lawlor D.A., Selvin E., Stampfer M. (2010). Emerging risk factors collaboration diabetes mellitus, fasting blood glucose concentration, and risk of vascular disease: A collaborative meta-analysis of 102 prospective studies. Lancet.

[2] Abchir O., Daoui O., Nour H., Yamari I., Elkhattabi S., Errougui A., Chtita S. (2023). Exploration of cannabis constituents as potential candidates against diabetes mellitus disease using molecular docking, dynamics simulations and Admet investigations. Sci. Afr..

[3] International Diabetes Federation (2021). Diabetes Federation. Diabetes around the world in 2021. IDF Diabetes Atlas.

[4] Fatima I., Taha M., Wadood A., Mohammad J.I., Khan H. (2018). 2-Aryl benzimidazoles: Synthesis, in vitro α-amylase inhibitory activity, and molecular docking study. Eur. J. Med. Chem..

[5] Tanaka M., Akiyama Y., Mori K., Hosaka I., Kato K., Endo K., Ogawa T., Sato T., Suzuki T., Yano T. (2024). Predictive modeling for the development of diabetes mellitus using key factors in various machine learning approaches. Diabetes Epidemiology Manag..

[6] Shadakshari A., Kumara T.S., Kumar N., Chandra S.J., Kumar K.A., Ramu R. (2023). Synthesis, characterization, and biocomputational assessment of the novel 3-hydroxy-4-(phenyl(pyridin-2-ylamino) methyl)-2-naphthoic acid derivatives as potential dual inhibitors of α-glucosidase and α-amylase enzymes. Results Chem..

[7] Gong L., Feng D., Wang T., Ren Y., Liu Y., Wang J. (2020). Inhibitors of α-amylase and α-glucosidase: Potential linkage for whole cereal foods on prevention of hyperglycemia. Food Sci. Nutr..

[8] Abchir O., Nour H., Daoui O., Yamari I., ElKhattabi S., El Kouali M., Talbi M., Errougui A., Chtita S. (2023). Structure-based virtual screening, ADMET analysis, and molecular dynamics simulation of Moroccan natural compounds as candidates for the SARS-CoV-2 inhibitors. Nat. Prod. Res..

[9] Lee S.-R., Choi J., Choi E.-K., Lee H., Han M., Ahn H.-J., Kwon S., Lee S.-W., Han K.-D., Oh S. (2023). Early rhythm control on diabetes-related complications and mortality in patients with type 2 diabetes mellitus and atrial fibrillation. Diabetes Res. Clin. Pract..

[10] Chaidam S., Saehlim N., Athipornchai A., Sirion U., Saeeng R. (2021). Synthesis and biological evaluation of 1,6-bis-triazole-2,3,4-tri-O-benzyl-α-d-glucopyranosides as a novel α-glucosidase inhibitor in the treatment of Type 2 diabetes. Bioorganic Med. Chem. Lett..

[11] Dahmani R., Manachou M., Belaidi S., Chtita S., Boughdiri S. (2021). Structural characterization and QSAR modeling of 1,2,4-triazole derivatives as α-glucosidase inhibitors. N. J. Chem..

[12] Fallah Z., Tajbakhsh M., Alikhani M., Larijani B., Faramarzi M.A., Hamedifar H., Mohammadi-Khanaposhtani M., Mahdavi M. (2022). A review on synthesis, mechanism of action, and structure-activity relationships of 1,2,3-triazole-based α-glucosidase inhibitors as promising anti-diabetic agents. J. Mol. Struct..

[13] Matin M.M., Matin P., Rahman R., Ben Hadda T., Almalki F.A., Mahmud S., Ghoneim M.M., Alruwaily M., Alshehri S. (2022). Triazoles and Their Derivatives: Chemistry, Synthesis, and Therapeutic Applications. Front. Mol. Biosci..

[14] Yeye E.O., Khan K.M., Chigurupati S., Wadood A., Rehman A.U., Perveen S., Maharajan M.K., Shamim S., Hameed S., Aboaba S.A. (2020). Syntheses, in vitro α-amylase and α-glucosidase dual inhibitory activities of 4-amino-1,2,4-triazole derivatives their molecular docking and kinetic studies. Bioorganic Med. Chem..

[15] Sharma P., Thakur A., Goyal A., Grewal A.S. (2023). Molecular docking, 2D-QSAR and ADMET studies of 4-sulfonyl-2-pyridone heterocycle as a potential glucokinase activator. Results Chem..

[16] Mitra S., Chatterjee S., Bose S., Panda P., Basak S., Ghosh N., Mandal S.C., Singhmura S., Halder A.K. (2024). Finding structural requirements of structurally diverse α-glucosidase and α-amylase inhibitors through validated and predictive 2D-QSAR and 3D-QSAR analyses. J. Mol. Graph. Model..

[17] Adeniji S.E., Uba S., Uzairu A. (2018). Theoretical modeling and molecular docking simulation for investigating and evaluating some active compounds as potent anti-tubercular agents against MTB CYP121 receptor. Futur. J. Pharm. Sci..

[18] Huang X., Ma S., Wu Y., Wan C., Zhao C., Wang H., Ju S. (2023). High-Throughput Screening of Amorphous Polymers with High Intrinsic Thermal Conductivity via Automated Physical Feature Engineering. J. Mater. Chem. A.

[19] Mouhsin M., Abchir O., El Otmani F.S., Oumghar A.A., Oubenali M., Chtita S., Mbarki M., Gamouh A. (2023). Identification of novel NLRP3 inhibitors: A comprehensive approach using 2D-QSAR, molecular docking, molecular dynamics simulation and drug-likeness evaluation. Chem. Pap..

[20] Yang L., Wang Y., Hao W., Chang J., Pan Y., Li J., Wang H. (2020). Modeling pesticides toxicity to Sheepshead minnow using QSAR. Ecotoxicol. Environ. Saf..

[21] Papa E., Sangion A., Arnot J.A., Gramatica P. (2018). Development of human biotransformation QSARs and application for PBT assessment refinement. Food Chem. Toxicol..

[22] ChemOffice (2016). PerkinElmer Informatics. http://www.cambridgesoft.com.

[23] PaDEL-Descriptor Yap (2011). An open source software to calculate molecular descriptors and fingerprints. J. Comput. Chem..

[24] Gramatica P., Chirico N., Papa E., Cassani S., Kovarich S. (2013). QSARINS: A new software for the development, analysis, and validation of QSAR MLR models. J. Comput. Chem..

[25] Mauri A., Consonni V., Todeschini R. (2017). Molecular descriptors. Handbook of Computational Chemistry.

[26] Gramatica P., Cassani S., Roy P.P., Kovarich S., Yap C.W., Papa E. (2012). QSAR Modeling is not “Push a Button and Find a Correlation”: A Case Study of Toxicity of (Benzo-)triazoles on Algae. Mol. Inform..

[27] Nour H., Abchir O., Belaidi S., Qais F.A., Chtita S., Belaaouad S. (2021). 2D-QSAR and molecular docking studies of carbamate derivatives to discover novel potent anti-butyrylcholinesterase agents for Alzheimer’s disease treatment. Bull. Korean Chem. Soc..

[28] Nour H., Abchir O., Belaidi S., Chtita S. (2022). Research of new acetylcholinesterase inhibitors based on QSAR and molecular docking studies of benzene-based carbamate derivatives. Struct. Chem..

[29] Chirico N., Gramatica P. (2012). Real External Predictivity of QSAR Models. Part 2. New Intercomparable Thresholds for Different Validation Criteria and the Need for Scatter Plot Inspection. J. Chem. Inf. Model..

[30] Bennani F.E., Doudach L., Karrouchi K., El Rhayam Y., Rudd C.E., Ansar M., Faouzi M.E.A. (2023). 2D-QSAR study and design of novel pyrazole derivatives as an anticancer lead compound against A-549, MCF-7, HeLa, HepG-2, PaCa-2, DLD-1. Comput. Toxicol..

[31] Sun G., Zhang Y., Pei L., Lou Y., Mu Y., Yun J., Li F., Wang Y., Hao Z., Xi S. (2021). Chemometric QSAR modeling of acute oral toxicity of Polycyclic Aromatic Hydrocarbons (PAHs) to rat using simple 2D descriptors and interspecies toxicity modeling with mouse. Ecotoxicol. Environ. Saf..

[32] Gramatica P., Sangion A. (2016). A Historical Excursus on the Statistical Validation Parameters for QSAR Models: A Clarification Concerning Metrics and Terminology. J. Chem. Inf. Model..

[33] Nath A., Ojha P.K., Roy K. (2023). Computational modeling of aquatic toxicity of polychlorinated naphthalenes (PCNs) employing 2D-QSAR and chemical read-across. Aquat. Toxicol..

[34] Gramatica P. (2007). Principles of QSAR models validation: Internal and external. QSAR Comb. Sci..

[35] Khedraoui M., Nour H., Yamari I., Abchir O., Errougui A., Chtita S. (2023). Design of a new potent Alzheimer’s disease inhibitor based on QSAR, molecular docking and molecular dynamics investigations. Chem. Phys. Impact.

[36] Eriksson L., Jaworska J., Worth A.P., Cronin M.T., McDowell R.M., Gramatica P. (2003). Methods for reliability and uncertainty assessment and for applicability evaluations of classification-and regression-based QSARs. Environ. Health Perspect..

[37] Netzeva T.I., Worth A.P., Aldenberg T., Benigni R., Cronin M.T., Gramatica P., Jaworska J.S., Kahn S., Klopman G., Marchant C.A. (2005). Current status of methods for defining the applicability domain of (quantitative) structure-activity relationships: The report and recommendations of ECVAM workshop 52. Altern. Lab. Anim..

[38] Yamari I., Abchir O., Siddique F., Zaki H., Errougui A., Talbi M., Bouachrine M., ElKouali M., Chtita S. (2024). The anticoagulant potential of Lippia Alba extract in inhibiting SARS-CoV-2 Mpro: Density functional calculation, molecular docking analysis, and molecular dynamics simulations. Sci. Afr..

[39] Ševčík J., Hostinová E., Solovicová A., Gašperík J., Dauter Z., Wilson K.S. (2006). Structure of the complex of a yeast glucoamylase with acarbose reveals the presence of a raw starch binding site on the catalytic domain. FEBS J..

[40] Guex N., Peitsch M.C. (1997). SWISS-MODEL and the Swiss-Pdb Viewer: An environment for comparative protein modeling. Electrophoresis.

[41] Trott O., Olson A. (2009). Software news and update AutoDock Vina: Improving the speed and accuracy of docking with a new scoring function, efficient optimization, and multithreading. J. Comput. Chem..

[42] Diniyah N., Alam B., Javed A., Alshammari F.H., Choi H.-J., Lee S.-H. (2023). In silico and docking studies on the binding activities of Keap1 of antioxidant compounds in non-oilseed legumes. Arab. J. Chem..

[43] Elangovan N., Sowrirajan S., Arumugam N., Almansour A.I., Mahalingam S.M., Kanchana S. (2023). Synthesis, solvent role (water and DMSO), antimicrobial activity, reactivity analysis, inter and intramolecular charge transfer, topology, and molecular docking studies on adenine derivative. J. Mol. Liq..

[44] Hanwell M.D., Curtis D.E., Lonie D.C., Vandermeersch T., Zurek E., Hutchison G.R. (2012). Avogadro: An advanced semantic chemical editor, visualization, and analysis platform. J. Cheminform..

[45] Liu X., Zang X., Yin X., Yang W., Huang J., Huang J., Yu C., Ke C., Hong Y. (2020). Semi-synthesis of C28-modified triterpene acid derivatives from maslinic acid or corosolic acid as potential α-glucosidase inhibitors. Bioorganic Chem..

[46] Morris G.M., Huey R., Lindstrom W., Sanner M.F., Belew R.K., Goodsell D.S., Olson A.J. (2009). AutoDock4 and AutoDockTools4: Automated docking with selective receptor flexibility. J. Comput. Chem..

[47] Yusuf T.L., Waziri I., Olofinsan K.A., Akintemi E.O., Hosten E.C., Muller A.J. (2023). Evaluating the in vitro antidiabetic, antibacterial and antioxidant properties of copper(II) Schiff base complexes: An experimental and computational studies. J. Mol. Liq..

[48] Shukla R., Munjal N.S., Singh T.R. (2019). Identification of novel small molecules against GSK3β for Alzheimer’s disease using chemoinformatics approach. J. Mol. Graph. Model..

[49] Yamari I., Abchir O., Nour H., El Kouali M., Chtita S. (2023). Identification of new dihydrophenanthrene derivatives as promising anti-SARS-CoV-2 drugs through in silico investigations. Main Group Chem..

[50] Duchowicz P.R., Talevi A., Bellera C., Bruno-Blanch L.E., Castro E.A. (2007). Application of descriptors based on Lipinski’s rules in the QSPR study of aqueous solubilities. Bioorganic Med. Chem..

[51] Chagas C.M., Moss S., Alisaraie L. (2018). Drug metabolites and their effects on the development of adverse reactions: Revisiting Lipinski’s Rule of Five. Int. J. Pharm..

[52] Lambring C.B., Fiadjoe H., Behera S.K., Basha R. (2024). Docking and molecular dynamic simulations of Mithramycin-A and Tolfenamic acid against Sp1 and survivin. Process. Biochem..

[53] Basnet S., Ghimire M.P., Lamichhane T.R., Adhikari R., Adhikari A. (2023). Identification of potential human pancreatic α-amylase inhibitors from natural products by molecular docking, MM/GBSA calculations, MD simulations, and ADMET analysis. PLoS ONE.

[54] Roos K., Wu C., Damm W., Reboul M., Stevenson J.M., Lu C., Dahlgren M.K., Mondal S., Chen W., Wang L. (2019). OPLS3e: Extending Force Field Coverage for Drug-Like Small Molecules. J. Chem. Theory Comput..

[55] Protein Preparation Wizard. https://www.schrodinger.com/science-articles/protein-preparation-wizard.

[56] Mark P., Nilsson L. (2001). Structure and Dynamics of the TIP3P, SPC, and SPC/E Water Models at 298 K. J. Phys. Chem. A.

[57] Uba A.I., Chea J., Hoag H., Hryb M., Bui-Linh C., Wu C. (2022). Binding of a positive allosteric modulator CDPPB to metabotropic glutamate receptor type 5 (mGluR5) probed by all-atom molecular dynamics simulations. Life Sci..

[58] D.E. Shaw Research (2021). Desmond Molecular Dynamics System, Maestro-Desmond Interoperability Tools.

[59] Bhattacharya P., Abualnaja K.M., Javed S. (2023). Theoretical studies, spectroscopic investigation, molecular docking, molecular dynamics and MMGBSA calculations with 2-hydrazinoquinoline. J. Mol. Struct..

